# A case of giardiasis in a Japanese organic farmer

**DOI:** 10.1002/jgf2.357

**Published:** 2020-06-28

**Authors:** Daiki Yokokawa, Kiyoshi Shikino, Masatomi Ikusaka

**Affiliations:** ^1^ Department of General Medicine Chiba University Hospital Chiba City Japan

**Keywords:** farmer, giardiasis trophozoite, organic agriculture, zoonosis

## Abstract

A 61‐year‐old Japanese man presented with a 2‐month history of colicky abdominal pain and watery diarrhea. He had begun organic farming using cattle manure 1 year previously.

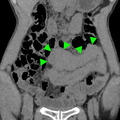

A 61‐year‐old Japanese man presented with a 2‐month history of colicky abdominal pain and watery diarrhea. He had begun organic farming using cattle manure 1 year previously. His abdominal pain was colicky and relieved by defecation, the odor of which had changed. Physical examination revealed mild abdominal tenderness. Laboratory data showed low total protein (5.8 g/dL) and albumin (3.5 g/dL) without inflammation (C‐reacted protein 0.1 mg/dL). Abdominal computed tomography revealed small intestinal wall thickening (Figure 1). A *Giardia* duodenalis trophozoite was detected in a fecal smear (Figure 2 and Video S1), so giardiasis was diagnosed. Metronidazole (250 mg) three times daily for 10 days resolved symptoms completely.

**Figure 1:**
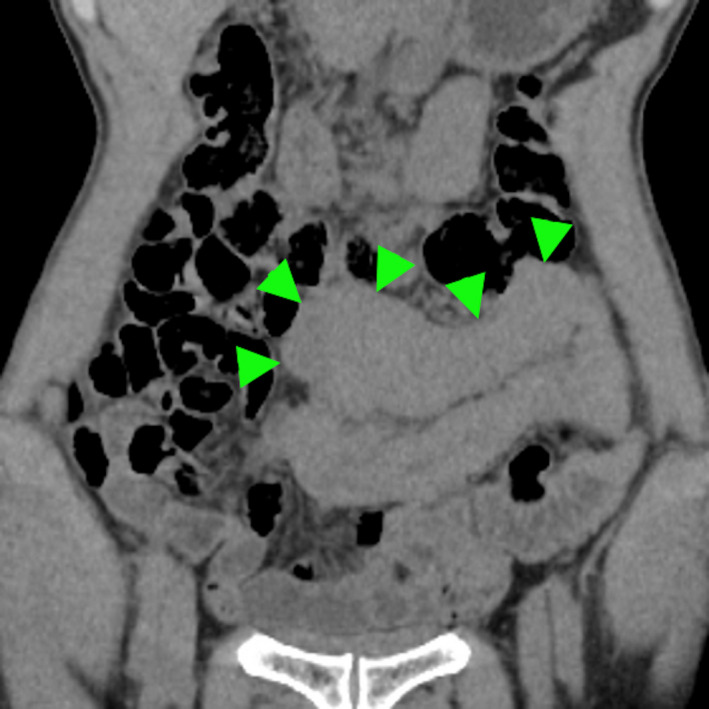
Abdominal computed tomography revealed small intestinal wall thickening (arrowheads)

**Figure 2:**
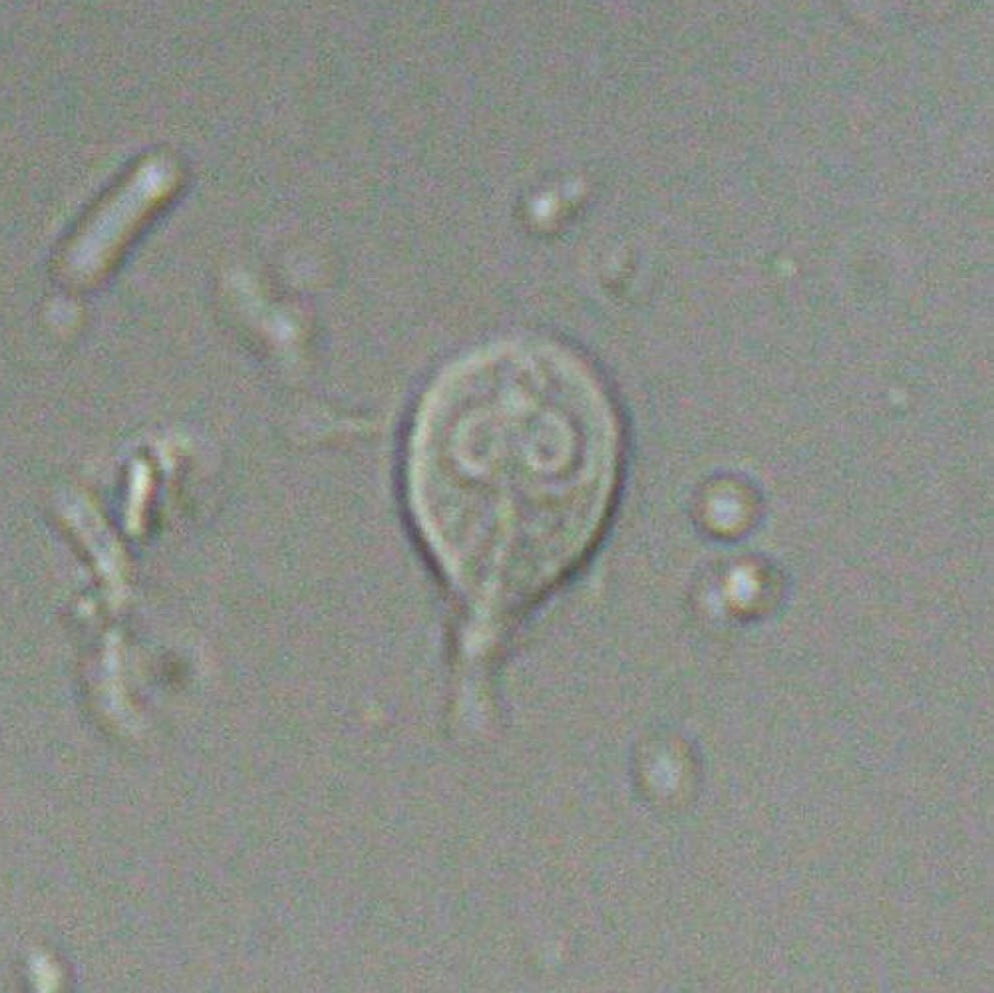
*Giardia* trophozoite in a fecal smear (direct smear). Image taken at 400 × magnification

Giardiasis is an intestinal infection caused by *G* duodenalis. Symptoms of acute giardiasis include watery diarrhea (90%), foul‐smelling fatty stools (75%), and abdominal cramps and bloating (71%).^1^ Giardiasis is zoonotically transmitted through *G* duodenalis cysts in feces of mammals like cattle, sheep, and dogs through the fecal‐oral route. Outbreaks often occur via contaminated water or food. In Japan, about 100 cases have been reported for public health surveillance, and an outbreak occurred in 2010.^2^ Half of these cases were exposed abroad; only 1% contracted *G* duodenalis through sewage water or feces. Transmission is often associated with farmers and veterinarians. Handwashing and glove use are important for preventing transmission.^3^ In symptomatic patients, antibiotic therapy with oral metronidazole for 5‐7 days is effective and simultaneously kills parasites in the stool.

## CONFLICT OF INTERESTS

The authors have stated explicitly that there are no conflicts of interest in connection with this article.

## AUTHOR CONTRIBUTIONS

All authors had access to the data and a role in writing the manuscript.

## INFORMED CONSENT

We have obtained written informed consent of the patient for publication.

## Supporting information

Video S1
